# Protective Effect of Resveratrol against IL-1β-Induced Inflammatory Response on Human Osteoarthritic Chondrocytes Partly via the TLR4/MyD88/NF-κB Signaling Pathway: An “*in Vitro* Study”

**DOI:** 10.3390/ijms15046925

**Published:** 2014-04-22

**Authors:** Li Liu, Hailun Gu, Huimin Liu, Yongliang Jiao, Keyu Li, Yue Zhao, Li An, Jun Yang

**Affiliations:** 1 Department of Nutrition and Food Hygiene, School of Public Health, China Medical University, No. 92 Beier Road, Heping District, Shenyang 110001, Liaoning, China; E-Mails: liuhuimin_@163.com (H.L.); jylaaa@126.com (Y.J.); lkylky@163.com (K.L.); zhao_yue@163.com (Y.Z.); anli@mail.cmu.edu.cn (L.A.); yangjun@mail.cmu.edu.cn (J.Y.); 2 Department of Orthopedics, Shengjing Hospital, China Medical University, No. 36 Sanhao Street, Sanhao District, Shenyang 110004, Liaoning, China; E-Mail: guhailun_@163.com

**Keywords:** resveratrol, toll-like receptor 4, nuclear factor-κB, chondrocytes, osteoarthritis, interleukin-1β, inflammation

## Abstract

Resveratrol is a natural polyphenolic compound that prevents inflammation in chondrocytes and animal models of osteoarthritis (OA) via yet to be defined mechanisms. The purpose of this study was to determine whether the protective effect of resveratrol on IL-1β-induced human articular chondrocytes was associated with the TLR4/MyD88/NF-κB signaling pathway by incubating human articular chondrocytes (harvested from osteoarthritis patients) with IL-1β before treatment with resveratrol. Cell viability was evaluated using the MTT (3-(4,5-dimethylthiazol-2-yl)-2,5-diphenyltetrazolium bromide) assay and TNFα levels in culture supernatants were measured by ELISA(Enzymelinked immunosorbent assay). The levels of TLR4 and its downstream signaling targets (MyD88 and TRAF6) and IL-1β were assessed by measuring the levels of mRNA and protein expression by real-time RT-PCR and western blot analysis, respectively, in addition to assessing NF-κB activation. In addition, TLR4 siRNA was used to block TLR4 expression in chondrocytes further demonstrating that resveratrol prevented IL-1β-mediated inflammation by TLR4 inhibition. We found that resveratrol prevented IL-1β-induced reduction in cell viability. Stimulation of chondrocytes with IL-1β caused a significant up-regulation of TLR4 and its downstream targets MyD88 and TRAF6 resulting in NF-κB activation associated with the synthesis of IL-1β and TNFα. These IL-1β-induced inflammatory responses were all effectively reversed by resveratrol. Furthermore, activation of NF-κB in chondrocytes treated with TLR4 siRNA was significantly attenuated, but not abolished, and exposure to resveratrol further reduced NF-κB translocation. These data suggested that resveratrol prevented IL-1β-induced inflammation in human articular chondrocytes at least in part by inhibiting the TLR4/MyD88/NF-κB signaling pathway suggesting that resveratrol has the potential to be used as a nutritional supplement to counteract OA symptoms.

## 1. Introduction

Osteoarthritis (OA) is a common chronic joint disease, particularly in the aging population, characterized primarily by articular cartilage degeneration and secondary bone hyperplasia [1]. Cartilage damage is the most important feature in OA. Cartilage provides mechanical stability and resistance to loads [2]. Chondrocytes represent the only cellular components of cartilage [3] that produce type II collagen and proteoglycan that constitute the cartilaginous matrix and play a dominant role in cartilage function [4].

The cause of OA involves injury, loss of cartilage structure and function, or a disturbance of proinflammatory and anti-inflammatory signaling pathways [5,6]. In addition, the integrity of the subchondral bone, synovium, menisci, ligaments, periarticular muscles and nerves also impact the pathogenesis of OA [7]. For example, synovial inflammation is directly linked to cartilage degradation, which further up-regulates mediators and molecules such as interleukin (IL)-8, IL-6, and inducible nitric oxide synthase (iNOS) [8]. To date there are no cures for OA since treatments resulting in the successful restoration of cartilage have not been developed [9]. Current OA treatment strategies involve reducing pain and inflammation, limiting the loss of functional capacity and maintaining joint mobility. Several *in vitro* and preclinical studies have suggested the protective roles of dietary polyphenols on progression of OA, in terms of alleviating chondrocyte inflammation and further cartilage damage/destruction, through their ability to directly or indirectly interact with the joint-associated tissues (*i.e*., articular cartilage, bone, or synovium), resulting in the mitigation of joint pain [8,10,11]. A recent study also reported that an extra-virgin olive oil diet (the beneficial effects of olive oil could be due to its phytochemicals) combined with physical activity exerted drastically anti-osteoarthritis effect by increasing lubricin and eliciting anti-inflammatory responses [12], suggesting that reducing inflammation by phytochemicals is beneficial to alleviating OA diseases.

Toll-like receptor 4 (TLR4) is a pattern recognition receptor that elicits inflammatory responses critical to the development of antigen-specific adaptive immune responses [13]. Recent studies have demonstrated that TLR4 induces inflammatory responses following the recognition of several endogenous ligands associated with tissue injury [14]. TLR4 is not only expressed by immune cells but also expressed by non-professional antigen presenting cells (such as cells comprising articular cartilage) [15] that possess functional properties similar to monocytes or macrophage [13,16,17]. Cellular activation following ligation of TLR4 can lead to increased production of IL-1β and reduced production of type II collagen and aggrecan [15]. In contrast, inhibition of TLR4 relieved up-regulation of IL-1β and interfered with OA progression [18]. A previous report demonstrated that TLR4 had a higher level of expression in isolated and cultured chondrocytes from OA patients than in those from non-OA patients [19] indicating that TLR4 may play an important role in OA pathogenesis. In addition, this study also determined that alarmins S100A8 and S100A9 which were associated with cartilage breakdown in human OA tissues exerted net catabolic effect on human chondrocytes in a TLR4-depent fashion [19], further indicating that TLR4 played a critical role in OA.

Resveratrol (*trans*-3,5,40-trihydroxystilbene) is a natural polyphenolic compound present in grapes, berries and peanuts [20]. Abundant evidence has demonstrated that resveratrol could be a candidate for OA therapy [21–23]. Resveratrol exerts antiosteoarthritis effects by mediating anti-apoptotic, anti-inflammatory, and anti-oxidative functions in chondrocytes (*in vitro*) and in animal models for OA [24–29]. The anti-inflammatory effects of resveratrol on chondrocytes have been linked to the inhibition of nuclear facor (NF)- κB which down-regulates pro-inflammatory gene products [30,31]. A previous report demonstrated that resveratrol suppressed NF-κB activation induced by TLR4-mediated signaling in RAW264.7 cells [32] and another report showed that resveratrol exerted the protective effects by inhibiting cell death and preventing inflammation in cardiomyocytes undergoing anoxia/reoxygenation, which might be associated with the TLR4/NF-κB signaling pathway [33]. However, it is not clear whether resveratrol can interfere with NF-κB by inhibiting TLR4 signaling in human inflammatory articular chondrocytes. Therefore, the aim of this study was to determine whether the protective effects of resveratrol on IL-1β-stimulated human articular chondrocytes involved signaling via TLR4 that in turn affected the myeloid differentiation factor 88 (MyD88)/NF-κB signaling pathway. We first determined whether resveratrol given at various concentrations exerted anti-inflammatory responses in IL-1β-stimulated human articular chondrocytes from OA patients. Second, we explored whether the protective effect, at least partly due to signaling via the TLR4/MyD88/NF-κB pathway.

## 2. Results and Discussion

The principal findings of the present study were: (i) no cell toxicity of resveratrol at various concentrations (6.25–200 μM) was detected in human articular chondrocytes from OA patients. In addition, a relative low resveratrol concentrations (6.25, 12.5 or 25 μM) promoted cell proliferation in contrast to higher concentrations (50, 100 or 200 μM) that had no effect; (ii) resveratrol exerted anti-inflammatory effects on IL-1β-stimulated human articular chondrocytes from OA patients in a dose-dependent manner (6.25–200 μM); and (iii) the anti-inflammatory effects of resveratrol were partly due to the inhibition of TLR4 expression associated with the subsequent block of the MyD88 dependent pathway that in turn interfered with of NF-κB activation.

### 2.1. Resveratrol Improved the Inhibition of IL-1β-Induced Chondrocyte Proliferation

**Figure 1.**
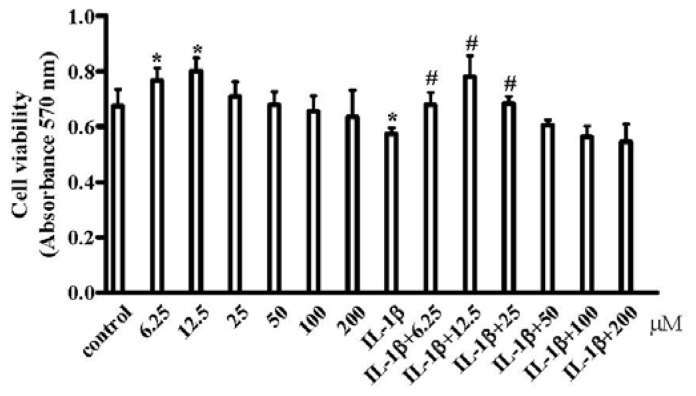
Effects of resveratrol and IL-1β on the viability and proliferation of chondrocytes *in vitro*. Serum-starved (0.5% FCS) human articular chondrocytes from OA patients were treated with various concentration of resveratrol (0, 6.25, 12.5, 25, 50, 100, 200 μM) only or in the presence of 10 ng/mL IL-1β for 24 h. Cell viability was examined using the MTT (3-(4,5-dimethylthiazol-2-yl)-2,5-diphenyltetrazolium bromide)method. Treatment groups were analyzed in triplicate and the data expressed as the mean ± SD from three independent experiments. *p* < 0.05, * *vs.* control, # *vs.* IL-1β.

Pro-inflammatory cytokines such as IL-1β and tumor necrosis factor alpha (TNFα) have been shown to mediate chondrocytes degradation associated with OA [34–37]. Therefore, to mimic an inflammatory response in chondrocytes during OA, chondrocytes were pretreated with IL-1β, a recognized inducer of OA *in vitro* [38] prior to treatment with various concentration of resveratrol. By using the MTT assay, we found that resveratrol (6.25–200 μM) had no discernable toxic effects on chondrocytes cultured in the presence or absence of IL-1β. Moreover, at 6.25 and 12.5 μM, resveratrol significantly stimulated chondrocyte proliferation. In addition, viability and proliferation of cells exposed to IL-1β (10 ng/mL) was significantly impaired but not in the presence of resveratrol (6.25 to 25 μM) (Figure 1) indicating that a relative low concentrations (6.25, 12.5 or 25 μM) could promote cell proliferation in contrast to higher concentrations (50, 100 or 200 μM).

### 2.2. Resveratrol Suppressed IL-1β-Induced TLR4 Expression and TNFα Production

Recent studies have indicated that resveratrol might be used to treat and prevent OA progression in an experimental animal model by preventing apoptosis and conferring anti-inflammatory and antioxidant properties via yet to be defined mechanisms [27,28,39]. TLR4 activation leads to translocation of NF-κB into the nucleus [40,41] resulting in the induction of inflammatory responses [13]. Resveratrol acting as an anti-inflammatory dietary phytochemical blocked some catabolic effects of proinflammatory mediators such as IL-1β and TNFα via the inhibition of NF-κB [20,42].

**Figure 2.**
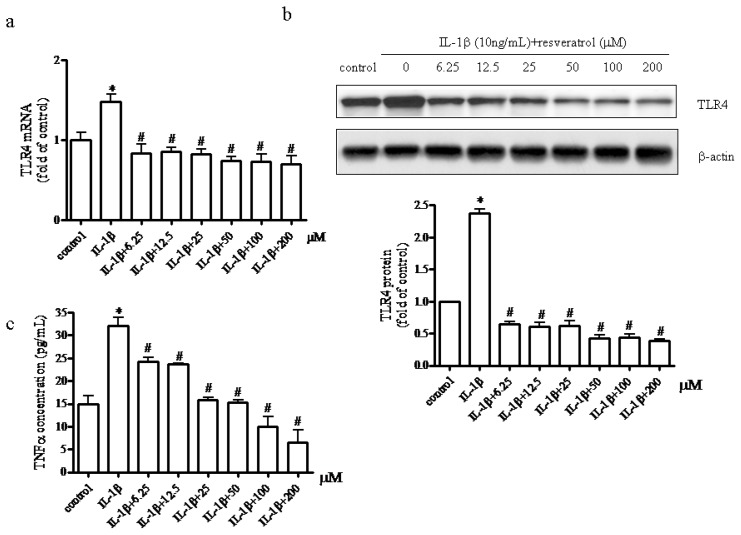
Effect of resveratrol on TLR4 mRNA and protein synthesis and TNFα production. (**a**) Serum-starved (0.5% FCS) human articular chondrocytes were treated with 10 ng/mL IL-1β alone for 1 h before being treated with different concentrations of resveratrol (0, 6.25, 12.5, 25, 50, 100, 200 μM) and 10 ng/mL IL-1β for 24 h. The relative expression levels of TLR4 mRNA were determined by real-time RT-PCR. *p* < 0.01, * *vs.* control, # *vs.* IL-1β; (**b**) Effect of resveratrol on TLR4 protein expression. After chondrocytes were incubated in the presence or absence of resveratrol as described above, whole cell protein concentrations were determined and the relative amount of TLR4 assessed by Western blot analysis. *p* < 0.01, * *vs.* control, # *vs.* IL-1β; (**c**) TNF-α concentrations in the culture supernatants were determined by ELISA. This assay was performed in triplicate and the data expressed as the mean ± SD from three independent experiments. *p* < 0.01, * *vs.* control, # *vs.* IL-1β.

To determine whether TLR4 was activated in the presence of IL-1β and whether resveratrol could inhibit the IL-1β-induced TLR4 activation we incubated chondrocytes with IL-1β (10 ng/mL) for 1 h followed by incubation in the presence or absence of different concentrations (6.25–200 μM) of resveratrol for 24 h. Using RT-PCR and western blot analysis, TLR-4 mRNA (Figure 2a) and protein expression levels (Figure 2b) were determined, demonstrating a marked increase in TLR4 mRNA and protein expression levels in chondrocytes treated with IL-1β. Discrepancies between our results and data presented by Chen *et al.* [43] that demonstrated that TLR4 expression was not affected by IL-1β may be due to different cell sources (in this study we used OA chondrocytes which may be more sensitive to response to IL-1β stimulation). However, our data were supported by Schelbergen *et al.* [19] that demonstrated that TLR4 mRNA was higher in OA chondrocytes than in non-OA chondrocytes. In addition, we demonstrated that TLR4 expression in chondrocytes treated with IL-1β and resveratrol (6.25 to 200 μM) was significantly decreased, suggesting that resveratrol exerted negative effect on TLR4 expression not only at a relatively small concentration (6.25 μM) but also a relatively big concentration (200 μM).

To address whether resveratrol at various concentration exerted anti-inflammatory effects on IL-1β-stimulated chondrocytes the TNFα expression profile in culture supernatants in the absence or presence of resveratrol was assessed. As shown in Figure 2c, 10 ng/mL IL-1β treatment significantly increased TNFα concentration. By contrast, resveratrol at the doses tested in this study reduced TNFα production in a dose-dependent manner implying that resveratrol could prevent IL-1β-induced chondrocytes damage by reducing TNFα production.

### 2.3. Resveratrol Suppressed Activation of the TLR4/NF-κB Signaling Pathway and Subsequent Synthesis of IL-1β in IL-1β-Stimulated Human Chondrocytes

We next investigated whether the protective effects of resveratrol on human chondrocytes involved the TLR4/NF-κB signaling pathway. In the presence of IL-1β resveratrol at both low (6.25–25 μM) and high (50–200 μM) concentrations had significant negative effects on TLR4 signaling although an increase in cellular proliferation was observed at the lower doses but not at the higher concentrations. Therefore, a representative low and high dose treatment of resveratrol (12.5 and 100 μM) was used to examine whether resveratrol could inhibit the IL-1β-induced activation of NF-κB and subsequent IL-1β synthesis.

**Figure 3.**
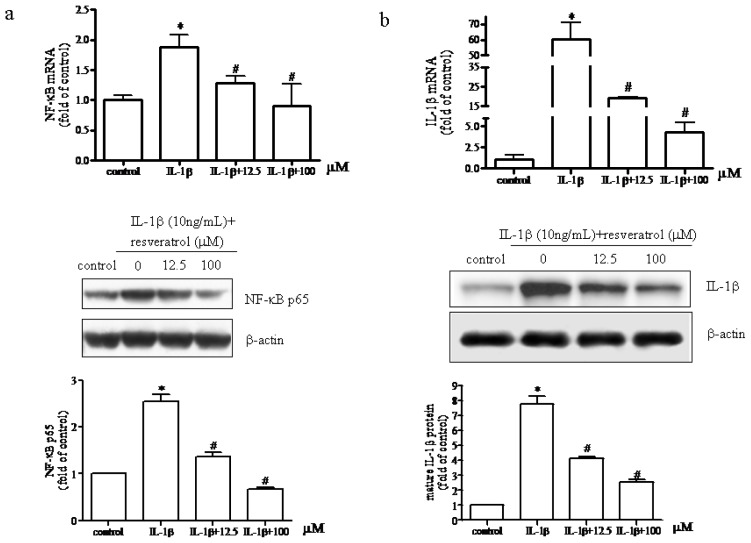
Effect of resveratrol on IL-1β-induced NF-κB activation and the levels of IL-1β mRNA and protein synthesis in human chondrocytes. (**a**) Serum-starved (0.5% FCS) human articular chondrocytes were treated with 10 ng/mL IL-1β for 1 h before the addition of 12.5 or 100 μM resveratrol for 24 h. Total chondrocyte RNA and nuclear protein concentrations were determined. The relative expression of NF-κB mRNA and protein was determined by real-time RT-PCR and western blot analysis. *p* < 0.05, * *vs.* control, # *vs.* IL-1β; (**b**) Effect of resveratrol on IL-1β mRNA and protein expression. After chondrocytes were cultured in the presence or absence of resveratrol as described above, total RNA and whole cell protein concentrations were determined by real-time RT-PCR and western blot analysis. *p* < 0.05, * *vs.* control, # *vs.* IL-1β.

As shown in Figure 3a, IL-1β (10 ng/mL) up-regulated NF-κB mRNA expression and nuclear translocation that was significantly inhibited by resveratrol (12.5 or 100 μM). These results suggested that resveratrol could reverse IL-1β-mediated effects on chondrocytes by suppressing NF-κB activation suggesting that the anti-inflammatory response conferred by resveratrol might be mediated in part by the TLR4/NF-κB pathway. We also examined the expression levels of IL-1β mRNA and protein in chondrocytes. Western blot analyses designed to measure active IL-1β in chondrocytes supported the above observations that IL-1b activated NF-κB and that increases in IL-1β mRNA and protein synthesis could be significantly reversed by resveratrol (Figure 3b). Resveratrol (12.5 or 100 μM) significantly reduced the production of mature IL-1β. These results indicated that in IL-1β-stimulated human chondrocytes cultured in the presence of either low (12.5 μM) or high (100 μM) resveratrol concentrations blocked inflammatory responses by inhibiting the TLR4/NF-κB signaling pathway.

### 2.4. TLR4-Knockdown Increased the Effects of Resveratrol on IL-1β-Stimulated Human Chondrocytes

**Figure 4.**
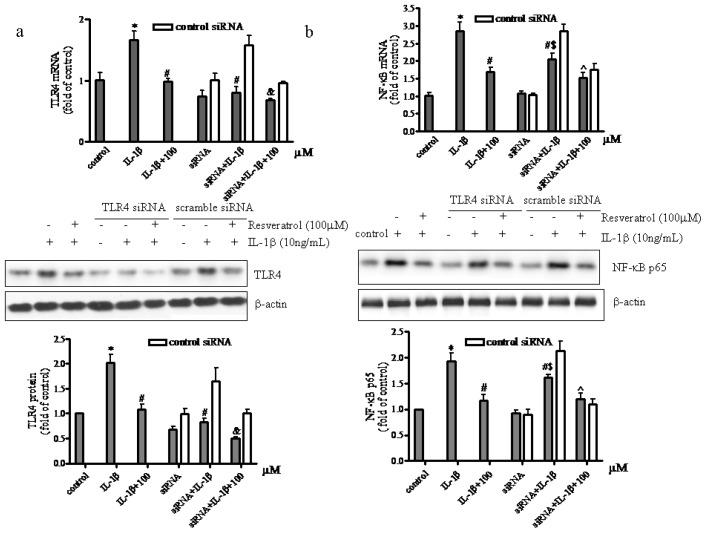
Effect of resveratrol on TLR4 and NF-κB expression in IL-1β-treated human articular chondrocytes after TLR4-knockdown. (**a**) Serum-starved (0.5% FCS) articular chondrocytes were pretreated with TLR4 siRNA or scrambled siRNA and then exposed to 10 ng/mL IL-1β for 1 h followed by the addition of 100 μM resveratrol and 10 ng/mL IL-1β for 24 h. Expression of TLR4 mRNA and protein was determined by real-time RT-PCR and western blot analysis, respectively. *p* < 0.01, * *vs.* control, # *vs.* IL-1β, and & *vs.* IL-1β + 100; (**b**) After incubation of chondrocytes as described above, total RNA and nuclear protein concentrations were determined by real-time RT-PCR and western blot analysis, respectively. *p* < 0.01, * *vs.* control, # *vs.* IL-1β, $ *vs.* siRNA, ^ *vs.* siRNA +IL-1β.

To further confirm that the TLR4/NF-κB signaling pathway in human articular chondrocytes was affected by resveratrol TLR4 siRNA was used to block TLR4 expression followed by analysis of NF-κB activation. Administration of TLR4 siRNA reduced TLR4 mRNA levels by approximately 30% (Figure 4a).

Greater inhibition effects of the TLR4 siRNA were not observed probably because: (i) the transfection and inhibition efficiency of TLR4 shRNA plasmid we used in chondrocytes perhaps was somewhat low, although it may be suitable for a variety of cell lines; (ii) the basal level of TLR4 mRNA in chondrocytes was low, so the inhibitory effects of the TLR4 siRNA were less effective. Although the downregulation of TLR4 mRNA by TLR4 siRNA was not significant, the subsequent results demonstrated that TLR4 siRNA still exert inhibition effect. Results illustrated in Figure 4a showed a significant up-regulation of TLR4 mRNA and protein in IL-1β-treated chondrocytes, whereas in chondrocytes receiving both IL-1β and resveratrol, a significant reduction of TLR4 expression was observed compared to IL-1β-treated chondrocytes. Conversely, the addition of IL-1β failed to increase TLR4 expression levels in TLR4 siRNA pretreated chondrocytes. In addition, compared to chondrocytes cultured in the presence of IL-1β and resveratrol, chondrocytes pretreated with TLR4 siRNA presented with a marked reduction in TLR4 expression in the presence of IL-1β and resveratrol. Data presented in Figure 4b demonstrated that NF-κB mRNA and protein expression in IL-1β-treated chondrocytes was activated significantly, whereas in chondrocytes receiving both IL-1β and resveratrol, a significant reduction of NF-κB expression was observed compared to IL-1β-treated chondrocytes. In chondrocytes pretreated with TLR4 siRNA NF-κB activation was significantly attenuated, but not abolished, indicating that in IL-1β-induced chondrocytes TLR4 could be activated and that activation of NF-κB could be mediated by TLR4 stimulation. These data further suggested that activation of NF-κB was not only due to TLR4 stimulation but also involved other mechanisms. Interestingly, the addition of resveratrol to chondrocytes treated with both TLR4 siRNA and IL-1β had an even greater reduction in NF-κB expression levels. This finding confirmed that resveratrol might exert anti-inflammatory effects on chondrocytes, at least in part via the TLR4/NF-κB pathway.

### 2.5. Effect of Resveratrol on MyD88 and TRAF6 Expression in IL-1β-Stimulated Human Chondrocytes

**Figure 5.**
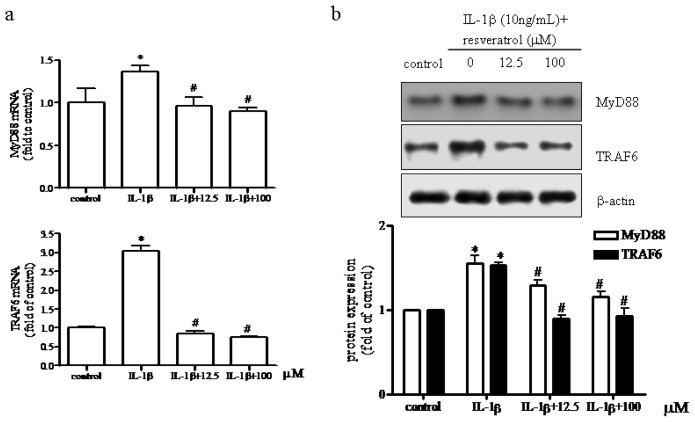
Effect of resveratrol on the TLR4 signaling pathway in human chondrocytes. The effect of resveratrol on (**a**) MyD88 and (**b**) TRAF6 mRNA and protein expression was assessed in serum-starved (0.5% FCS) articular chondrocytes treated with 10 ng/mL IL-1β for 1 h before being treated with either 12.5 or 100 μM resveratrol for 24 h. The relative expression levels of MyD88 and TRAF6 mRNA were determined by real-time RT-PCR. *p* < 0.05, * *vs.* control, # *vs.* IL-1β. After incubation of chondrocytes in the presence or absence of resveratrol as described above, the relative amount of MyD88 and TRAF6 were determined by western blot analysis.

The classic TLR4 signaling pathway involves both MyD88-dependent pathway and independent pathways (that involves signaling via TRIF (TIRdomain-containing adaptor-inducing interferon-β)). Several reports have indicated that resveratrol specifically targeted the TRIF complex of the TLR4 signaling pathway in different cell types [44,45]. We therefore examined in particular whether the MyD88-dependent pathway was involved in human chondrocytes. To test this idea, we measured the expression of MyD88 and TNF receptor-associated factor (TRAF) 6 mRNA and protein following treatment with IL-1β in the presence of either 12.5 or 100 μM resveratrol. As shown in Figure 5a,b, IL-1β alone increased MyD88 protein expression by 55% and the presence of either 12.5 or 100 μM resveratrol decreased MyD88 expression by 33% and 40%, respectively. TRAF6 expression was significantly up-regulated after IL-1β treatment, whereas the addition of resveratrol reduced TRAF6 expression compared to IL-1β alone. These data confirmed that the Myd88-dependent signaling pathway was initiated in IL-1β-treated chondrocytes exposed to resveratrol supporting data presented by Sebai *et al.* that demonstrated that resveratrol treatment prevented LPS-induced extracellular lipoperoxidation of AR42J cells (in part due to signaling via the MyD88 pathway) [46]. However, our data did not exclude the involvement of the MyD88 independent (TRIF) signaling pathway suggesting that in our model resveratrol acted in a MyD88-dependent manner that may or may not have also activated the TRIF-dependent pathway in human articular chondrocytes.

**Figure 6.**
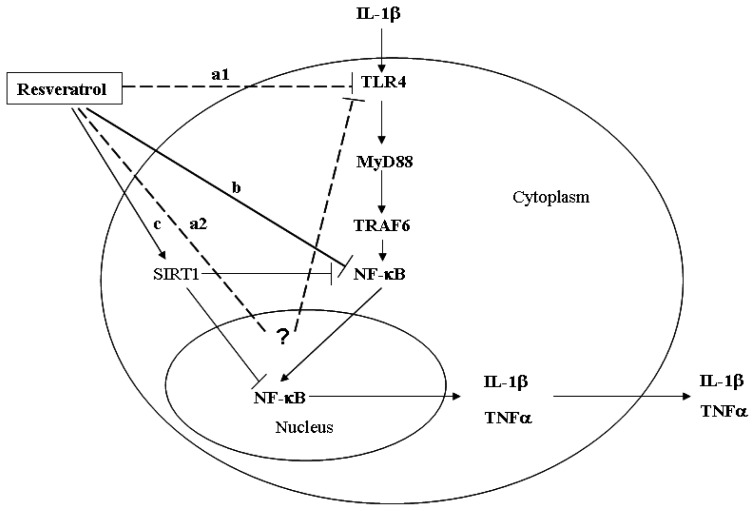
Schematic describing the proposed anti-inflammatory response resulting from resveratrol treatment and its effects on TLR4 following stimulation of chondrocytes with IL-1β. IL-1β-upregulated TLR4 expression results in the activation of the TLR4/MyD88/NF-κB signaling pathway causing translocation of NF-κB into the nucleus resulting in the production of proinflammatory cytokines such as IL-1β and TNFα. Resveratrol-mediated directly inhibition of TLR4 expression could block the TLR4/MyD88/NF-κB signaling pathway (a1, dashed line), or resveratrol could affect TLR4 pathway processes in the nucleus through unknown regulatory mechanisms that regulate the gene expression (a2, dashed line); Direct inhibition of NF-κB by resveratrol, as described by Csaki [25] and Shakibaei [26] is shown in (b, solid line); Mechanisms involved in NF-κB suppression by resveratrol-activated SIRT1, as described by Lei [31] are shown in (c, solid line).

Data presented in this report provided evidence suggesting that the protective effects of resveratrol were mediated in part due to activation of the TLR4/MyD88/NF-κB signaling pathway. A schematic of potential molecular mechanisms involved is described in Figure 6, suggesting that resveratrol treatment inhibited IL-1β-induced inflammatory responses in chondrocytes. However, due to the preliminary results and *in vitro* nature of the study, future studies conducted *in vivo* will be required to determine the efficacy, safety, and molecules targets affected by resveratrol using OA animal models.

## 3. Experimental Section

### 3.1. Reagents and Antibodies

Collagenase type II, MTT, resveratrol, and the protease inhibitor cocktail were all purchased from Sigma-Aldrich (St. Louis, MO, USA). Dulbecco’s modified Eagle’s medium (DMEM)/Ham’s F-12 (1:1) and fetal calf serum (FCS) were obtained from Hyclone (Thermo Scientific, Logan, UT, USA). Resveratrol was prepared as a 400 mM solution in DMSO and then further diluted in cell culture medium (the final concentration of DMSO in medium was not more than 0.1%). IL-1β was purchased from PeproTech (Rocky Hill, NJ, USA). Enzymelinked immunosorbent assay (ELISA) kit was obtained from Boster Biotechnology (Wuhan, China). RNAiso plus and the RT-PCR kit were purchased from TaKaRa (Dalian, China). The BSA kit was obtained from Bio-Rad (Hercules, CA, USA). Polyclonal anti-β-actin (sc-477787), anti-TLR4 (sc-293072), anti-IL-1β (sc-7884), anti-MyD88 (sc-74532), and anti-TRAF6 (sc-8409) antibodies and TLR4 siRNA (sc-40261) were purchased from Santa Cruz Biotechnology (Santa Cruz, CA, USA). The antibody specific for phosporylated NF-κB p65 (No. 3033) was obtained from Cell Signal Technology (Beverly, MA, USA). Anti-mouse and anti-rabbit secondary antibodies were obtained from Pierce (Rockford, IL, USA) and the enhanced chemiluminescence reagent was purchased from Amersham Biosciences (Buckinghamshire, UK).

### 3.2. Chondrocyte Isolation and Culture

Human knee articular chondrocytes were isolated from 30 patients (ages 48–70 years old) undergoing joint replacement surgery for osteoarthritis. The study was approved by the Ethics Committee at the Shengjing Hospital China Medical University, Shenyang, China and informed consent was obtained from all participants. Cartilage slices were digested primarily with 0.25% typsin for 1 h at 37 °C and subsequently with 0.04% collagenase type II overnight in a 37 °C water bath. Primary cells cultures were seeded at 1~2 × 10^5^ cells/mL in a 25 cm^2^ flask at 37 °C, 5% CO_2_ in DMEM/F12 medium supplemented with 10% FCS, penicillin (100 U/mL) and streptomycin (100 U/mL). All chondrocytes were used for the described experiments at the third passage.

### 3.3. Cell Viability Assay

Chondrocytes (5000/well) were seeded in triplicate in a 96-well plate and cultured for 24 h in the presence or absence of 10 ng/mL IL-1β for 1 h followed by the addition of various resveratrol concentrations (0, 6.25, 12.5, 25, 50, 100, 200 μM) for 24 h at 37 °C, 5% CO_2_. Cell viability and proliferation was determined using the MTT method. Briefly, cells were washed with PBS 3 times and then MTT (5 mg/mL) was added to each well and incubated for 4 h at 37 °C 5% CO_2_. The culture medium was then removed and replaced with an equal volume of DMSO to dissolve blue formazan crystals. Absorbance was determined at 570 nm using a micro plate reader (Bio-Rad, Hercules, CA, USA) and data expressed as the mean ± standard deviation (SD) of three independent experiments.

### 3.4. Chondrocyte Treatment

Chondrocytes were washed three times with PBS and incubated for 1 h in serum-starved media (0.5% FCS). Serum-starved chondrocytes were pre-stimulated as follows: 10 ng/mL IL-1β for 1 h before the addition of various concentrations of resveratrol (0–200 μM) for 24 h, or 10 ng/mL IL-1β for 1 h before the addition of either 12.5 or 100 μM resveratrol for 24 h. TLR4 small interference (si) RNA was used to knock down TLR4 expression followed by exposure to 10 ng/mL IL-1β for 1 h before being treated with 100 μM resveratrol for 24 h.

### 3.5. TLR4 siRNA Treatment

A modified siRNA transfection protocol (Santa Cruz Biotechnology, Paso Robles, CA, USA) was used to transfect 1 × 10^6^ cells with 1.0 μg TLR4 siRNA or a scramble siRNA sequence that served as a negative control. After transfection, cells were cultured for additional 48 h in DMEM/F12 medium containing 10% FCS. Culture medium was then replaced with serum starved media (0.5% FCS) and exposed to 10 ng/mL IL-1β for 1 h before being the addition of 100 μM resveratrol for 24 h. The following chondrocyte treatment groups were included (1) control; (2) IL-1β; (3) IL-1β + 100 μM resveratrol; (4) TLR4 siRNA; (5) TLR4 siRNA + IL-1β; (6) TLR4 siRNA+IL-1β + 100 μM resveratrol; (7) control siRNA; (8) control siRNA + IL-1β; and (9) control siRNA+IL-1β + 100 μM resveratrol.

### 3.6. RNA Extraction and Reverse Transcription Polymerase Chain Reaction (RT-PCR)

Total RNA was extracted using RNAiso plus according to the manufacturer’s instructions. The PCR reactions of amplified TLR4, MyD88, TRAF6, NF-κB, and IL-1β products were quantitatively measured using the 7500 real time PCR detection system (ABI, Carlsbad, CA, USA). Each PCR reaction mixture contained 10 μL of 2× SYBR Green Master Mix (TaKaRa, Dalian, China), 0.8 μL of forward and or reverse primers (10 μmol/μL), 0.4 μL of Rox Reference DyeII (50×) (TaKaRa, Dalian, China) and 2 μL of cDNA. Forty cycles with denaturation at 95 °C for 5 s and an annealing and extension temperature of 60 °C for 34 s in a total volume of 20 μL were used to amplify the respective targets. Amplification of β-actin was used as endogenous control gene to analyze the real-time PCR data of target genes and data analyzed using the 2^−ΔΔ^*^C^*^t^ Method [47]. The relative level of target gene mRNA was calculated by subtracting *C*_t_ values of the control gene from the *C*_t_ values of the targets. The sequences of the forward (F) and reverse (R) primers are listed 5′ to 3′ as follows: β-actin (101 bp) (F) 5′-CACACTGTGCCCATCTACGA-3′, (R) 5′-CTCAGTGAGGATCTTCATGA GGTAGT-3′. TLR4 (148 bp) (F) 5′-AGGACTGGGTAAGGAATGAGC-3′, (R) 5′-ATCACCTTT CGGCTTTTATGG-3′. NF-κB (218 bp) (F) 5′-AGGAGAGGATGAAGGAGTTGTG-3′, (R) 5′-CCAGAGTAGCCCAGTTTTTGTC-3′. IL-1β (247 bp) (F) 5′-AGTGGCAATGAGGAT GACTTGT-3′, (R) 5′-AGATGAAGGGAAAGAAGGTGCT-3′. MyD88 (178 bp) (F) 5′-CACTCAG CCTCTCTCCAGGT-3′, (R) 5′-AGTCTTCAGGGCAGGGACA-3′. TRAF6 (191 bp) (F) 5′-CTGG AAGCCCTAAGACAAAGA-3′, (R) 5′-GGCAAGGAAAGGCACTGTT-3′.

### 3.7. Protein Extraction and Immunoblot Analysis

Whole cell lysates and chondrocyte nuclear extracts were prepared and subjected to SDS-PAGE. Briefly, chondrocytes were washed with ice cold PBS and whole cell proteins were extracted with lysis buffer (50 mM Tris-HCl, pH 7.5, 0.1 mM Na_3_VO_4_, 1% Nonidet P-40, 25 mM NaF, 2 mM EDTA, 2 mM EGTA, 1 mM DTT, and 1% protease inhibitor cocktail) on ice for 30 min. Cell debris was removed by centrifugation and supernatants stored at −80 °C until use. For isolation of chondrocyte nuclei, chondrocytes were trypsinized and washed 3 times with PBS. Supernatants were carefully removed by centrifugation and the cell pellet re-suspended in hypotonic lysis buffer containing a protease inhibitor cocktail and incubated on ice for 20 min followed by the addition of 10% NP-40. The cell suspension was then mixed vigorously for 20 s and then centrifuged. Nuclear extraction buffer was then added to the pellets and incubated for 30 min with intermittent mixing. Extracts were centrifuged and the supernatant (nuclear extracts) stored at −80 °C until use.

The protein concentrations of the whole cell and nuclear extracts were determined using a BSA kit. Protein samples were boiled for 5 min in 1× SDS sample buffer (50 mM Tris-HCl, pH 6.8, 20% glycerol, 2% SDS, 0.02% bromophenol blue) containing 2%-mercaptoethanol. Extracted proteins subjected to SDS-PAGE and then transferred onto a polyvinylidene difluoride membrane for 3 h at 4 °C. Respective membranes were then blocked with 5% fat-free milk for 1 h at room temperature, washed 3 times with Tween-Tris buffered saline and then incubated with the following primary antibodies: anti-TLR4, anti-MyD88, anti-TRAF6, anti-IL-1β, (1:300 dilution), or anti-phosphorylated NF-κB p65 (1:1000 dilution) overnight at 4 °C. The next day the blots were washed and incubated in the presence of horseradish peroxidase-conjugated secondary antibodies (1:5000 dilution) for 45 min at room temperature. The enhanced chemiluminescence reagent was used to identify reactive bands that were quantified using the Scion Image 4.0 software (Scion Corporation, Frederick, MD, USA).

### 3.8. ELISA Assay

TNFα concentrations in the culture supernatants were measured using a commercial enzyme-linked immunosorbent assay (ELISA) kit (Boster Biotechnology, Wuhan, China) according to the manufacture’s protocol.

### 3.9. Statistical Analysis

All data were expressed as the mean ± SD. Statistical analysis were performed using one-way analysis of variance (ANOVA) using SPSS 13.0 software (SPSS Inc., Chicago, IL, USA). *p* < 0.05 was considered to be statistically significant.

## 4. Conclusions

Our results demonstrated that TLR4 played an important role in the pathogenesis of OA and that resveratrol exerted anti-inflammatory effects on IL-1β-induced human articular chondrocytes at least in part by inhibiting the TLR4/MyD88/NF-κB signaling pathway. Our findings also indicated that resveratrol could protect chondrocytes against the development of OA suggesting that resveratrol could be developed as a novel therapeutic agent for the treatment of OA. Currently, there are no effective treatments to cure OA and current available therapies such as non-steroid anti-inflammatory drugs only alleviate symptoms and are associated with adverse side-effects. Resveratrol is a dietary polyphenol nutritional supplement with the potential of counteracting symptoms associated with OA. Therefore, additional studies should be carried out to determine whether other TLR4 signaling pathways might be involved in the pathogenesis of OA and better define the mechanism by which resveratrol interfered with the TLR4/NF-κB pathway, that is, either directly or through other regulatory mechanisms. Additional *in vivo* studies will be required to understand the efficacy, safety, and molecules targeted by resveratrol followed by clinical trials to evaluate the effects of resveratrol on OA.

## References

[1] Busija L., Bridgett L., Williams S.R., Osborne R.H., Buchbinder R., March L., Fransen M. (2010). Osteoarthritis. Best Pract. Res. Clin. Rheumatol.

[2] Campo G.M., Avenoso A., Campo S., D’Ascola A., Nastasi G., Calatroni A. (2010). Molecular size hyaluronan differently modulates toll-like receptor-4 in LPS-induced inflammation in mouse chondrocytes. Biochimie.

[3] Chiu Y.C., Yang R.S., Hsieh K.H., Fong Y.C., Way T.D., Lee T.S., Wu H.C., Fu W.M., Tang C.H. (2007). Stromal cell-derived factor-1 induces matrix metalloprotease-13 expression in human chondrocytes. Mol. Pharmacol.

[4] Umlauf D., Frank S., Pap T., Bertrand J. (2010). Cartilage biology, pathology, and repair. Cell Mol. Life Sci.

[5] Sofat N., Ejindu V., Kiely P. (2011). What makes osteoarthritis painful? The evidence for local and central pain processing. Rheumatology (Oxford).

[6] Goldring M.B., Otero M. (2011). Inflammation in osteoarthritis. Curr. Opin. Rheumatol.

[7] Man G., Mologhianu G. (2014). Osteoarthritis pathogenesis—A complex process that involves the entire joint. J. Med. Life.

[8] Henrotin Y., Lambert C., Couchourel D., Ripoll C., Chiotelli E. (2011). Nutraceuticals: Do they represent a new era in the management of osteoarthritis?—A narrative review from the lessons taken with five products. Osteoarthr. Cartil.

[9] Sgaglione N.A. (2005). Biologic approaches to articular cartilage surgery: Future trends. Orthop. Clin. N. Am.

[10] Henrotin Y., Kurz B., Aigner T. (2005). Oxygen and reactive oxygen species in cartilage degradation: Friends or foes?. Osteoarthr. Cartil.

[11] Henrotin Y., Kurz B. (2007). Antioxidant to treat osteoarthritis: Dream or reality?. Curr. Drug Targets.

[12] Musumeci G., Trovato F.M., Pichler K., Weinberg A.M., Loreto C., Castrogiovanni P. (2013). Extra-virgin olive oil diet and mild physical activity prevent cartilage degeneration in an osteoarthritis model: An *in vivo* and *in vitro* study on lubricin expression. J. Nutr. Biochem.

[13] Takeda K., Kaisho T., Akira S. (2003). Toll-like receptors. Annu. Rev. Immunol.

[14] Miyake K. (2007). Innate immune sensing of pathogens and danger signals by cell surface Toll-like receptors. Semin. Immunol.

[15] Bobacz K., Sunk I.G., Hofstaetter J.G., Amoyo L., Toma C.D., Akira S., Weichhart T., Saemann M., Smolen J.S. (2007). Toll-like receptors and chondrocytes: The lipopolysaccharide-induced decrease in cartilage matrix synthesis is dependent on the presence of toll-like receptor 4 and antagonized by bone morphogenetic protein 7. Arthritis Rheumatol.

[16] Hoshino K., Takeuchi O., Kawai T., Sanjo H., Ogawa T., Takeda Y., Takeda K., Akira S. (1999). Cutting edge: Toll-like receptor 4 (TLR4)-deficient mice are hyporesponsive to lipopolysaccharide: Evidence for TLR4 as the Lps gene product. J. Immunol.

[17] Loniewski K.J., Patial S., Parameswaran N. (2007). Sensitivity of TLR4- and -7-induced NF kappa B1 p105-TPL2-ERK pathway to TNF-receptor-associated-factor-6 revealed by RNAi in mouse macrophages. Mol. Immunol.

[18] Abdollahi-Roodsaz S., Joosten L.A., Roelofs M.F., Radstake T.R., Matera G., Popa C., van der Meer J.W., Netea M.G., van den Berg W.B. (2007). Inhibition of Toll-like receptor 4 breaks the inflammatory loop in autoimmune destructive arthritis. Arthritis Rheumatol.

[19] Schelbergen R.F., Blom A.B., van den Bosch M.H., Sloetjes A., Abdollahi-Roodsaz S., Schreurs B.W., Mort J.S., Vogl T., Roth J., van den Berg W.B. (2012). Alarmins S100A8 and S100A9 elicit a catabolic effect in human osteoarthritic chondrocytes that is dependent on Toll-like receptor 4. Arthritis Rheumatol.

[20] Kundu J.K., Shin Y.K., Kim S.H., Surh Y.J. (2006). Resveratrol inhibits phorbol ester-induced expression of COX-2 and activation of NF-kappaB in mouse skin by blocking IkappaB kinase activity. Carcinogenesis.

[21] Dave M., Attur M., Palmer G., Al-Mussawir H.E., Kennish L., Patel J., Abramson S.B. (2008). The antioxidant resveratrol protects against chondrocyte apoptosis via effects on mitochondrial polarization and ATP production. Arthritis Rheumatol.

[22] Csaki C., Mobasheri A., Shakibaei M. (2009). Synergistic chondroprotective effects of curcumin and resveratrol in human articular chondrocytes: Inhibition of IL-1beta-induced NF-kappaB-mediated inflammation and apoptosis. Arthritis Res. Ther.

[23] Shakibaei M., Csaki C., Nebrich S., Mobasheri A. (2008). Resveratrol suppresses interleukin-1beta-induced inflammatory signaling and apoptosis in human articular chondrocytes: Potential for use as a novel nutraceutical for the treatment of osteoarthritis. Biochem. Pharmacol.

[24] Eo S.H., Cho H., Kim S.J. (2013). Resveratrol inhibits nitric oxide-induced apoptosis via the NF-kappa B pathway in rabbit articular chondrocytes. Biomol. Ther. (Seoul).

[25] Csaki C., Keshishzadeh N., Fischer K., Shakibaei M. (2008). Regulation of inflammation signalling by resveratrol in human chondrocytes *in vitro*. Biochem. Pharmacol..

[26] Shakibaei M., John T., Seifarth C., Mobasheri A. (2007). Resveratrol inhibits IL-1 beta-induced stimulation of caspase-3 and cleavage of PARP in human articular chondrocytes *in vitro*. Ann. N. Y. Acad. Sci..

[27] Elmali N., Esenkaya I., Harma A., Ertem K., Turkoz Y., Mizrak B. (2005). Effect of resveratrol in experimental osteoarthritis in rabbits. Inflamm. Res.

[28] Elmali N., Baysal O., Harma A., Esenkaya I., Mizrak B. (2007). Effects of resveratrol in inflammatory arthritis. Inflammation.

[29] Im H.J., Li X., Chen D., Yan D., Kim J., Ellman M.B., Stein G.S., Cole B., Kc R., Cs-Szabo G. (2012). Biological effects of the plant-derived polyphenol resveratrol in human articular cartilage and chondrosarcoma cells. J. Cell Physiol.

[30] Manna S.K., Mukhopadhyay A., Aggarwal B.B. (2000). Resveratrol suppresses TNF-induced activation of nuclear transcription factors NF-kappa B, activator protein-1, and apoptosis: potential role of reactive oxygen intermediates and lipid peroxidation. J. Immunol.

[31] Lei M., Wang J.G., Xiao D.M., Fan M., Wang D.P., Xiong J.Y., Chen Y., Ding Y., Liu S.L. (2012). Resveratrol inhibits interleukin 1beta-mediated inducible nitric oxide synthase expression in articular chondrocytes by activating SIRT1 and thereby suppressing nuclear factor-kappaB activity. Eur. J. Pharmacol.

[32] Youn H.S., Lee J.Y., Fitzgerald K.A., Young H.A., Akira S., Hwang D.H. (2005). Specific inhibition of MyD88-independent signaling pathways of TLR3 and TLR4 by resveratrol: Molecular targets are TBK1 and RIP1 in TRIF complex. J. Immunol.

[33] Zhang C., Lin G., Wan W., Li X., Zeng B., Yang B., Huang C. (2012). Resveratrol, a polyphenol phytoalexin, protects cardiomyocytes against anoxia/reoxygenation injury via the TLR4/NF-kappaB signaling pathway. Int. J. Mol. Med.

[34] Blanco F.J., Guitian R., Moreno J., de Toro F.J., Galdo F. (1999). Effect of antiinflammatory drugs on COX-1 and COX-2 activity in human articular chondrocytes. J. Rheumatol.

[35] Hashimoto S., Ochs R.L., Komiya S., Lotz M. (1998). Linkage of chondrocyte apoptosis and cartilage degradation in human osteoarthritis. Arthritis Rheumatol.

[36] Heraud F., Heraud A., Harmand M.F. (2000). Apoptosis in normal and osteoarthritic human articular cartilage. Ann. Rheum. Dis.

[37] Aigner T., Kim H.A. (2002). Apoptosis and cellular vitality: Issues in osteoarthritic cartilage degeneration. Arthritis Rheumatol.

[38] Iacono A., Gomez R., Sperry J., Conde J., Bianco G., Meli R., Gomez-Reino J.J., Smith A.B., Gualillo O. (2010). Effect of oleocanthal and its derivatives on inflammatory response induced by lipopolysaccharide in a murine chondrocyte cell line. Arthritis Rheumatol.

[39] De Oliveira R.M., Pais T.F., Outeiro T.F. (2010). Sirtuins: Common targets in aging and in neurodegeneration. Curr. Drug Targets.

[40] Akira S., Takeda K. (2004). Toll-like receptor signalling. Nat. Rev. Immunol.

[41] Akira S., Takeda K., Kaisho T. (2001). Toll-like receptors: Critical proteins linking innate and acquired immunity. Nat. Immunol.

[42] Estrov Z., Shishodia S., Faderl S., Harris D., Van Q., Kantarjian H.M., Talpazm M., Aggarwal B.B. (2003). Resveratrol blocks interleukin-1beta-induced activation of the nuclear transcription factor NF-kappaB, inhibits proliferation, causes S-phase arrest, and induces apoptosis of acute myeloid leukemia cells. Blood.

[43] Chen Y.J., Tsai K.S., Chiu C.Y., Yang T.H., Lin T.H., Fu W.M., Chen C.F., Yang R.S., Liu S.H. (2013). EGb761 inhibits inflammatory responses in human chondrocytes and shows chondroprotection in osteoarthritic rat knee. J. Orthop. Res.

[44] She Q.B., Bode A.M., Ma W.Y., Chen N.Y., Dong Z. (2001). Resveratrol-induced activation of p53 and apoptosis is mediated by extracellular-signal-regulated protein kinases and p38 kinase. Cancer Res.

[45] Jung D.Y., Lee H., Jung B.Y., Ock J., Lee M.S., Lee W.H., Suk K. (2005). TLR4, but not TLR2, signals autoregulatory apoptosis of cultured microglia: A critical role of IFN-beta as a decision maker. J. Immunol.

[46] Sebai H., Ristorcelli E., Sbarra V., Hovsepian S., Fayet G., Aouani E., Lombardo D. (2010). Protective effect of resveratrol against LPS-induced extracellular lipoperoxidation in AR42J cells partly via a Myd88-dependent signaling pathway. Arch. Biochem. Biophys.

[47] Livak K.J., Schmittgen T.D. (2001). Analysis of relative gene expression data using real-time quantitative PCR and the 2(-Delta Delta C(T)) Method. Methods.

